# The evidence and role of music therapy in addressing the resilience of adolescents with eating disorders in the context of an acute inpatient admission

**DOI:** 10.1186/2050-2974-1-S1-O16

**Published:** 2013-11-14

**Authors:** Sarah Punch

**Affiliations:** 1 Monash Health, Australia

## 

Engaging in music is an integral part of identity formation and coping during adolescence. For a population who are establishing their place in the world, music serves as a medium for peer interaction, social acceptance and connectedness (McFerran, 2010). The effectiveness of music therapy with the adolescent population has been well documented (Baker, McFerran-Skewes & Krout, 2011). In particular, the use of familiar and unfamiliar songs provides a powerful platform for adolescents to express and share parts of their identity, communicate their feelings, as well as discover hope and guidance.

Drawing on the evidence, this paper will outline the ways that a music therapy program strives to improve coping for adolescents with an eating disorder within an acute paediatric inpatient setting and once discharged. This will be highlighted through case vignettes and patient perspectives.

The music therapy program outlined in this paper is an integral part of the multidisciplinary treating team and works closely alongside medical and nursing staff to address the needs of young people with eating disorders.

This abstract was presented in the **Care in Inpatient and Community Settings** stream of the 2013 ANZAED Conference.

